# Mendelian randomization studies in atopic dermatitis: causal insights across omics layers

**DOI:** 10.3389/fimmu.2026.1717812

**Published:** 2026-02-26

**Authors:** Alexandra Chera, Octavian Bucur, Roxana-Silvia Bumbăcea

**Affiliations:** 1 Department of Allergology, Carol Davila University of Medicine and Pharmacy, Bucharest, Romania; 2 Department of Allergology and Clinical Immunology, ‘Dr. Carol Davila’ Clinical Nephrology Hospital, Bucharest, Romania; 3 Genomics Research and Development Institute, Bucharest, Romania; 4 Viron Molecular Medicine Institute, Boston, MA, United States

**Keywords:** atopic dermatitis, Mendelian randomization, GWAS, causal inference, precision medicine, multi-omics, genomics, proteomics

## Abstract

Atopic dermatitis (AD) is a chronic inflammatory skin disease, shaped by genetic, immune and environmental factors. Even though this complex interaction has been thoroughly studied, uncovering causal relationships between specific exposures and AD remains challenging. Mendelian randomization (MR) has emerged as a powerful tool for establishing causal inferences between exposures and outcomes, using genome-wide association data. MR studies have provided evidence for potential causal associations between AD and a broad spectrum of traits and comorbidities, including neuropsychiatric, cardiometabolic, oncologic, immune-mediated conditions, as well as ophthalmologic and infectious complications. Moreover, multi-omic MR approaches have enabled biomarker and therapeutic target discovery, highlighting opportunities for screening refinement, drug repurposing, and precision medicine. By integrating causal inference tools within multiple omics layers, MR is reshaping our understanding of AD, accelerating progress toward precision medicine in immune-mediated diseases.

## 1 Introduction

Atopic dermatitis (AD) is a chronic inflammatory cutaneous disease characterized by intense pruritus, associated with papulo-vesicular lesions. The clinical presentation of AD is highly heterogeneous, since the morphology, distribution and evolution of the cutaneous lesions vary with age and ethnicity (1, 2). The worldwide prevalence of AD is 13% in children and 5% in adults (3, 4). The first symptoms of AD tend to appear in early infancy, 50% of the cases occurring in the first 6 months, but the vast majority of the cases tend to resolve spontaneously before adolescence. However, in some cases, the affliction persists into adulthood, generating high levels of discomfort and affecting the quality of life. Unfortunately, adult onset AD is on the rise (5, 6). Most patients develop high levels of total immunoglobulin E (IgE), as well as multiple allergen sensitization, while their skin is frequently colonized with *Staphylococcus aureus*, which has a contribution to AD pathogenesis (5, 7).

The association between AD and many different comorbidities, both allergic, such as allergic rhinitis (AR), asthma, or food allergies, and non-allergic (inflammatory diseases, psychological disorders) (2, 8, 9) has been identified and thoroughly studied. Both familial and genetic studies suggest a common susceptibility for atopic diseases, including AD (10, 11).

**Figure 1:**
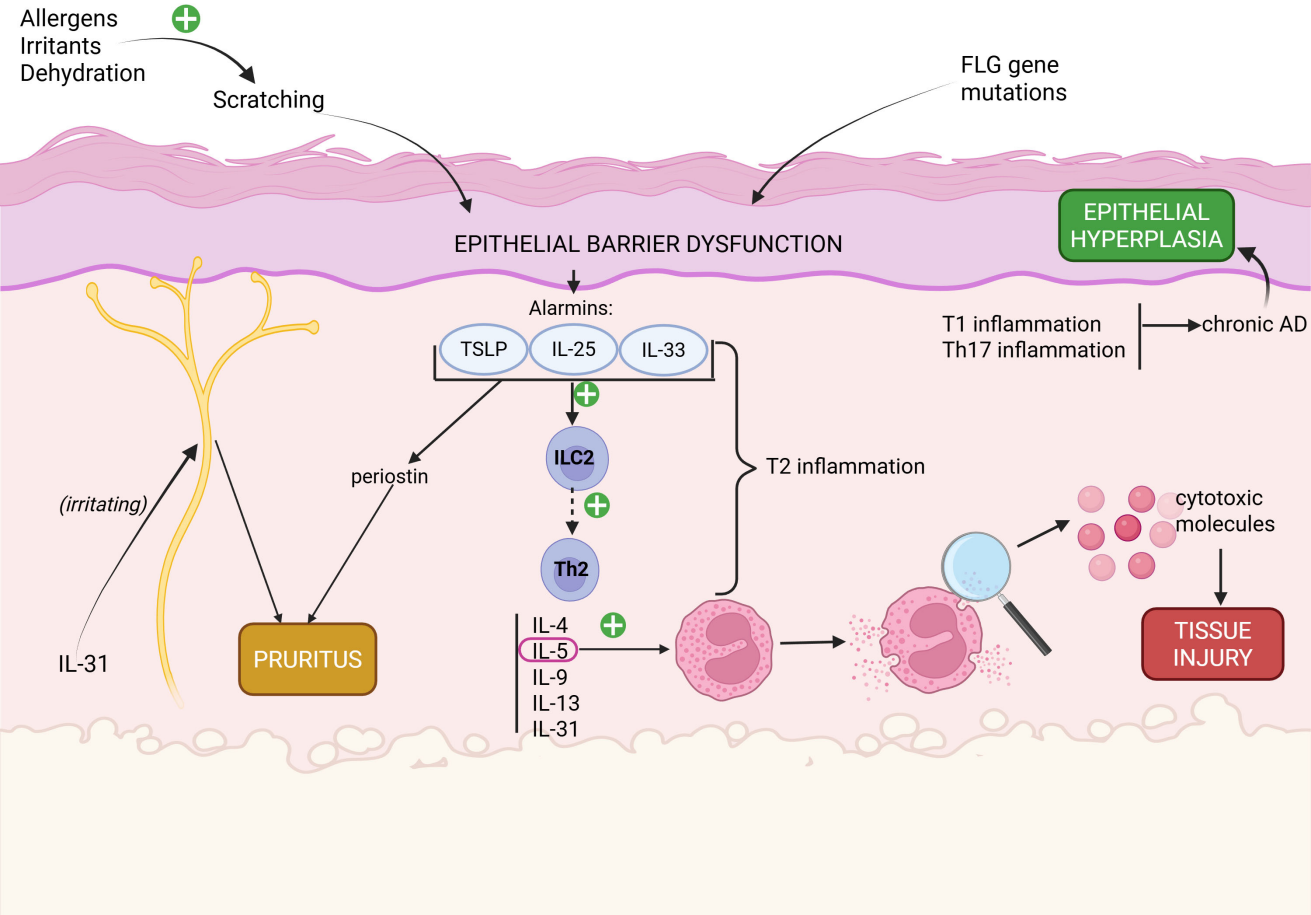
The pathogenesis of AD: the roles of epithelial barrier, innate and adaptive immunity, major cytokines, pruritus and aggravating factors. Created in BioRender. Chera, A. (2026) https://BioRender.com/tjortrn.

AD has a very complex and multifaceted pathogenesis, which involves interactions between genetic, biological (immunological) and environmental factors. A fundamental element in the pathogenesis of AD is the epithelial barrier dysfunction, caused either by a mutation of the FLG gene, and/or physically, through scratching, due to the inference of allergens, irritants or dehydration, all of which are able to trigger pruritus. The epithelial barrier dysfunction promotes the synthesis of alarmins,which trigger the type 2 inflammatory response, resulting in high concentrations of IL-4, IL-5 and IL-13.These interleukins are able to irritate the cutaneous free nerve endings, resulting in pruritus through direct stimulation of the specific receptors, while also suppressing keratinocyte differentiation. IL-5 promotes eosinophil proliferation, differentiation and activation of eosinophils, the latter leading to degranulation and release of cytotoxic molecules that contribute to the tissue injury. The chronic evolution of AD also involves types Th1 and Th17 of inflammation, ultimately leading to epidermal hyperplasia. (**Figure 1**) (1, 12).

Genome-wide association studies (GWAS) have identified over 100 loci associated with AD, confirming the involvement of skin barrier dysfunction and type 2 inflammation in the etiopathogenesis of AD, while also pointing toward autoimmunity and epidermal differentiation. However, some of these loci and the single nucleotide polymorphisms (SNPs) pertaining to them are located in intergenic regions with uncertain function, therefore their biological effects remain incompletely understood (13). This highlights the value of integrative approaches that combine deoxyribonucleic acid (DNA) variation with transcriptomic, proteomic, and metabolomic data (14).

Observational findings have indicated potential associations between AD and different comorbidities, such as asthma, AR, obesity, and depression. However, establishing causality in these instances is challenging, due to confounding factors and reverse causation. In order to overcome these difficulties, the analytical approach of Mendelian randomization (MR) becomes a viable option, as it uses genetic variants as instrumental variables, strengthening causal inferences (15). MR studies might also become useful in guiding multi-omics integration, since causal insights from genetic instruments can help in prioritizing molecular pathways and biomarkers for a more in-depth exploration. This combined strategy is expected to improve biological treatment optimization and patient stratification, especially since biologic therapies are increasingly used in AD, but currently require better molecular tools for guiding clinical use (14).

This narrative review aims to provide a broad descriptive mapping of MR studies in atopic dermatitis, and was designed to complement previous published work, such as (15), by substantially expanding the coverage to include 121 MR studies, and by encompassing a broader spectrum of clinical, biological, environmental, and translational contexts in which causal inference for atopic dermatitis has been explored. Formal methodological appraisal of Mendelian randomization assumptions and statistical validation of individual studies were outside the scope of the present work.

## 2 Genome-wide association studies in atopic dermatitis

The analysis of genetic variants has become possible through GWAS, which tend to focus on SNPs, uncovering the loci at which these occur, in order to identify specific phenotypic traits that may be associated with an increased risk for complex diseases (2, 16). By comparing the frequency of millions of variants between large cohorts of cases and controls, GWAS are able to identify loci that are associated with disease. However, these statistical associations only provide limited insight into the underlying mechanisms of diseases (13). Multiomics approaches have emerged in order to overcome this gap, enabling the prioritization of causal variants, clarification of regulatory effects, and identification of potential therapeutic targets (14).

In AD, GWAS have managed to reveal that genetic risk is driven by immune dysregulation and defects in the skin barrier (12). We have summarized the most relevant information from 21 GWAS that have identified novel susceptibility loci for AD after consulting the GWAS catalog online (17) in **Table 1** (18–28). The current knowledge reveals over 100 different loci involved in the etiopathogenesis of AD. Related studies were not included if they did not discover new AD loci.

**Table 1:** Genome-wide association studies of atopic dermatitis (AD).

First author (year)	Sample size (cases/controls)	Age group	Ancestry	Key findings: lead SNPs and genes	Effect estimates
Esparza-Gordillo et al. (2009) (18)	Discovery groups: - Set 1 = 939 cases of AD; 975 controls - Set 2 = 529 individuals (270 families) Follow-up groups: - Set 3 = 1,363 cases, 2739 controls - Set 4 = 1,274 cases, 1,218 controls	Adults and children	European (Germany, Czech Republic, Poland)	1 novel SNP: - rs7927894 (allele A), 11q13.5, gene: C11orf30 (EMSY)	- OR = 1.22, p=7.6×10^-10^ - approx. 13% Europeans are homozygous for rs7927894[A]
Sun et al. (2011) (19)	- 1,012 cases - 1,362 controls Replication study: - Chinese: 3,624 cases/12,197 controls - German: 1,806 cases/3,256 controls	Adults	Chinese Han, European (Germany)	2 novel loci: 1. 5q22.1, rs7701890, genes: TMEM232/SLC25A46 2. 20q13.33, rs6010620, genes: TNFRSF6B/ZGPAT	1. OR = 1.24, p=3.15×10^-9^ 2. OR = 1.17, p=3.0×10^-8^
Paternoster et al. (2012) (20)	- 5,606 cases - 20,565 controls (meta-analysis of GWAS)	Adults and children	European, North- American, Australian	3 novel loci: 1. 11q13.1, rs479844, gene: OVOL1 2. 19p13.2, rs2164983, gene: ACTL9 3. 5q31.1, rs2897442, gene: RAD500/IL13/KIF3A	1. OR = 0.88, p=1.1×10^−13^ 2. OR = 1.16, p=7.1×10^−9^ 3. OR = 1.11, p=3.8×10^−8^ - genes linked to epidermal proliferation and differentiation (OVOL1, ACTL9), as well as immune and autoimmune disorders (KIF3A)
Hirota (2012) (21)	- 3,328 cases - 14,992 controls	Adults	Japanese	8 novel SNPs: 1. rs13015714, 2q12, gene: IL1RL1-IL18R1-IL18RAP 2. rs176095, 6p21.3, gene: GPSM3 (MHC region) 3. rs878860, 11p15.4, gene: OR10A3-NLRP10 4. rs6780220, 3p21.33, gene: GLB1 5. rs12634229, 3q13.2, gene: CCDC80 6. rs4722404, 7p22, gene: CARD11 7. rs10995251, 10q21.2, gene: ZNF365 8. rs16999165, 20q13, gene: CYP24A1-PFDN4	1. OR = 1.27, p=8.36×10^−18^ 2. OR = 1.40, p=8.38×10^−20^ 3. OR = 1.32, p=1.95×10^−13^ 4. OR = 1.25, p=2.77×10^−16^ 5. OR = 1.29, p=1.56×10^−19^ 6. OR = 1.18, p=7.83×10^−9^ 7. OR = 1.28, p=5.85×10^−20^ 8. OR = 1.19, p=1.65×10^−8^ - genes linked to epidermal barrier functions, adaptive immunity, IL-1 signaling, apoptosis, inflammatory response, regulatory T cells and the vitamin D pathway.
Weidinger et al. (2013) (22)	- 1,563 cases - 4,054 controls Replication study: - 2,286 cases - 3,160 controls	Adults	European (United Kingdom, Ireland, Germany, Sweden)	4 novel loci, with the lead SNPs: 1. 1q21, rs11205006, gene: EDC/FLG 2. 11q13, rs2155219, gene: LRRC32 3. 5q31, rs2158177, gene: RAD50/IL13 4. 6p21, rs6474 (discovery) and rs2844509 (replication), MHC region	1. established risk locus, not re-tested in this study 2. OR = 1.32, p=1.61×10^−12^ 3. OR = 0.70, p=5.90×10^−11^ 4. OR = 1.31, p=4.33×10^−12^ - overlap between AD and psoriasis
Ellinghaus et al. (2014) (23)	- 2,425 cases - 5,449 controls	Adults	European (Germany, Ireland), Japanese, Chinese	4 novel SNPs: 1. rs17389644, 4q27, gene: IL2/IL21 2. rs12295535, 11p13, gene: PRR5L 3. rs2041733, 16p13.13, gene: CLEC16A/DEXI 4. rs16948048, 17q21.32, gene: ZNF652	1. OR = 1.19, p=1.39×10^−8^ 2. OR = 1.68, p=7.96×10^−13^ 3. OR = 1.23, p=3.44×10^−15^ 4. OR = 1.17, p=2.92×10^−9^
Paternoster et al. (2015) (24)	- 21,399 cases - 95,464 controls	Adults and children	European, North- American, Australian, Japanese, African, Latino	10 novel loci: 1. 1q21.3, rs61813875, gene: LCE3E 2. 2p13.3, rs112111458, gene: CD207 3. 2q12.1, rs12478877, gene: IL18R1/IL18RAP 4. 5p13.2, rs10214237, gene: IL7R 5. 5q31.1, rs12188917, gene: RAD50/IL13 6. 6p21.32, rs4713555, gene: HLA-DQ/DR 7. 11q13.1, rs7127307, gene: OVOL1 8. 11q13.5, rs2212434, gene: LRRC32 9. 14q11.2, rs2038255, gene: TRA (T-cell receptor alpha locus) 10. 20q13.33, rs6010620, gene: ADAMTS20	1. OR = 1.21, p=2.1×10^−21^ 2. OR = 1.19, p=3.0×10^−9^ 3. OR = 1.15, p=3.5×10^−10^ 4. OR = 1.16, p=1.1×10^−9^ 5. OR = 1.19, p=7.3×10^−10^ 6. OR = 1.21, p=3.8×10^−11^ 7. OR = 1.17, p=4.2×10^−11^ 8. OR = 1.18, p=6.1×10^−12^ 9. OR = 1.15, p=4.6×10^−10^ 10. OR = 1.1, p=2.4×10^−9^ - an 11th SNP was identified (rs7512552 - MRPS21), but it is a secondary signal in the FLG/EDC locus - genes linked to (auto)immune regulation (innate immune signaling, T cell activation) - substantial genetic overlap with other inflammatory and autoimmune diseases.
Schaarschmidt et al. (2015) (25)	- 924 cases - 5,506 controls Replication study: - 1,383 cases - 1,728 controls	Adults	European (Germany)	2 novel loci: 1. 2q24.3, rs6720763, gene: XIRP2 2. 9p21.3, rs10738626, gene: DMRTA1	1. OR = 1.29, p=4.37×10^−8^ 2. OR = 1.25, p=1.45×10^−8^ - replicated 5 of the 8 loci only reported in Asians (Hirota et al. (21))
Kim KW et al. (2015) (26)	- 246 cases (children with recalcitrant AD) - 551 controls (adults)	Children	Korean	5 novel loci: 1. 2p24.3, rs13403179, gene: NBAS 2. 6q22.33, rs675531, gene: THEMIS 3. 10p14, rs35766269, gene: GATA3 4. 13q21.31, rs9540294, gene: PCDH9 5. 15q24.3, rs3099143, gene: SCAPER	1. OR = 2.94, p=8.07×10^−8^ 2. OR = 2.19, p=6.82×10^−7^ 3. OR = 1.94, p=9.61×10^−8^ 4. OR = 2.65, p=1.01×10^−8^ 5. OR = 2.12, p=3.02×10^−7^ - 13q21.31 SNPs- significant association with serum total IgE value
Baurecht et al. (2015) (27)	- 2,262 AD cases - 4,489 psoriasis cases - 12,333 controls Data analyzed from different studies: - 2,425 AD cases - 3,580 psoriasis cases - 9,061 controls	Adults	European (Germany, Ireland)	2 novel loci with opposing effects in AD and psoriasis: 1. 2q31.2, rs62176107, gene: PRKRA 2. 5q33.1, rs17728338, gene: ANXA6/TNIP1	1. OR = 0.55, p=1.08×10^−34^ 2. OR = 0.70, p=3.96×10^−38^ - high degree of genomic coincidence between AD and psoriasis; - the opposing risk effects on AD and psoriasis suggest that same biological mechanisms might act differentially on AD vs psoriasis
Johansson et al. (2019) (28)	N=346,545 (after QC), out of which: - 7,884 AD cases - 239,773 controls (Asthma and hay fever cohorts also included; see original study for details)	Adults	European (United Kingdom)	- 1 novel AD locus (when analyzed separately): rs2485363, gene: TAGAP	- p=1.20×10^−8^ - 4 novel loci were also identified for the combined asthma-hay fever-AD phenotype - TAGAP has been previously associated with celiac disease and multiple sclerosis
Grosche et al. (2021) (29) (genome -wide meta- analysis)	- 20,016 cases - 38,0433 controls	Adults	European	3 exonic (functional) variants: 1. rs2431663, chromosome 5, gene: DUSP1 2. rs8192591, chromosome 6, gene: NOTCH4 3. rs61731289, chromosome 2, gene: SLC9A4	- identified through exonic/rare-variant analysis; no individual OR reported.
Tanaka et al. (2021) (30)	- 2639 cases - 115,648 controls	Adults	Japanese	8 novel loci: 1. rs80199341, chromosome 4, gene: AFF1 2. rs3757723, chromosome 7, gene: IFGB8 3. rs3125788, chromosome 9, gene: EHMT1 4. rs61865882, chromosome 10, gene: EGR2 5. rs6785012, chromosome 3, gene: ZBTB38 6. rs1444789, chromosome 10, gene: LOC105755953/ LOC101928272 7. rs56101042, chromosome 14, gene: TRAF3 8. rs11857092, chromosome 15, gene: IQGAP1	1. OR = 1.24, p=4.7×10^−8^ 2. OR = 1.33, p=1.5×10^−8^ 3. OR = 1.76, p=1.4×10^−8^ 4. OR = 0.75, p=2.6×10^−9^ 5. OR = 1.06, p=2.9×10^−9^ 6. OR = 1.07, p=2.7×10^−8^ 7. OR = 0.93, p=1.6×10^−8^ 8. OR = 0.94, p=4.5×10^−8^ - identified a shared polygenic architecture between Europeans and Asians
Sliz et al. (2022) (31) (genome-wide meta- analysis)	- 22,474 cases - 774,187 controls	Adults	European (Finland, Estonia, United Kingdom)	5 novel loci: 1. 3q24, rs150979174, gene: DIPK2A 2. 8q24.13, rs6996614, gene: TRIB1 3. 12q15, rs3947727, gene: IL22 4. 18q12.1, rs1361355315, gene: DSC1 5. 18q22.1, rs188720898, gene: SERPINB7	1. OR = 0.60, p=3.60×10^−8^ 2. OR = 1.08, p=1.55×10^−8^ 3. OR = 1.07, p=1.09×10^-11^ 4. OR = 1.54, p=3.53×10^−8^ 5. OR = 0.61, p=1.49×10^−9^ - DSC1, SERPINB7 - involved in protein folding, ensuring epidermal barrier stability
Margaritte‐Jeannin et al. (2022) (32)	- 1,208 cases with both asthma and AD - 7,599 controls	Adults and children	Caucasian (France, Germany, Austria, Switzerland, United Kingdom, Canada)	1 novel loci (2 SNPs) significantly associated with asthma-plus-eczema comorbidity: rs4778192 and rs2703978, 15q13, gene: OCA2.	- rs4778192: p=2.84×10^-7^ - rs2703978: p=2.98×10^−7^ - OCA2 variants: influence melanin synthesis, skin barrier integrity and immune functions; previously linked to pigmentation traits and melanoma.
Shirai et al. (2022) (33)	- 2,472 AD cases from the Japanese cohort - 12,285 self-reported AD cases from the European cohort - 433,663 controls (+712,767 controls for the replication study, which involved two new autoimmune diseases)	Adults	European (United Kingdom), Japanese	4 novel loci for allergic diseases (asthma, AD, pollinosis): 1. rs74052928, chromosome 1, gene: MIIP 2. rs575879774, chromosome 2, gene: CXCR4 3. rs7773622, chromosome 6, gene: SCML4 4. rs16902902, chromosome 8, gene: LINC00824 2 novel loci common for allergic and autoimmune diseases: 5. rs10803431, chromosome 1, gene: PRDM2 6. rs2053062, chromosome 5, gene: G3BP1	1. OR = 0.95, p=2.6×10^−9^ 2. OR = 1.05, p=3.4×10^−4^ 3. OR = 0.96, p=2.5×10^−7^ 4. OR = 0.94, p=2.1×10^−9^ 5. OR = 1.01, p=2.7×10^−5^ (through Cochrane’s Q test; all the rest - through the Lin-Sullivan method) 6. OR = 1.07, p=2.7×10^−8^ (only in the Japanese cohort) - shared genetics components were found between allergic (asthma, AD, pollinosis) and autoimmune (rheumatoid arthritis, Graves’ disease, type 1 diabetes mellitus, psoriasis and systemic lupus erythematosus) diseases.
Budu-Aggrey et al. (2023) (34)	European GWAS: - 60,653 cases - 864,982 controls Multi-ancestry GWAS: - 65,107 cases - 1,021,287 controls	Adults	European, North- American, Australian, Latino, African, Japanese	- 29 novel loci in the European-only analysis (chromosomes 1, 2, 4, 5, 6, 7, 8, 9, 11, 12, 14, 18; genes: RERE, TNFRSF1B, CSF1, CTSS, RORC, S100A7, S100A12, AHSA2P, MERTK, NEU4, IL15, OTULINL, LYRM7, RGS14, BACH2, CREB5, DOK2, AGO2, AQP3, NR4A3, MMP12, SIK3, PLXNC1, GPR132, PTPN2). - 3 novel loci in the multi-ancestry analysis: 1. rs9247, chromosome 2 2. rs34599047, chromosome 6 3. rs7773987, chromosome 6	1. p=1.92×10^−9^ 2. p=3.32×10^−8^ 3. p=1.22×10^−8^ (only found in the European and African cohorts) - the largest AD GWAS until 2023
Gautam et al. (2024) (35)	- 726 cases - 999 controls	Adults and children	African- American	- 2 novel loci specific for the African-American population: 1. 8q23.1, rs2195989, gene ANGPT1 2. 9p23, rs62538818, genes: LURAP1L/MPDZ (intergenic)	1. OR = 1.31, p=5.71×10^−4^ 2. OR = 1.77, p=1.22×10^−5^ - additional ancestry- specific signals (e.g., SGK1, EFR3A, MMP14) and prioritized genes (SLAIN2, RNF39, FOXA2). - ANGPT1 encodes angiopoietin-1, which regulates vascular development, inflammation, and tissue repair; SNP frequency: African>European - LURAP1L/MPDZ locus involved in cell signaling and epithelial barrier integrity; SNP frequency: European>African
Kim JW et al. (2024) (36)	- 636 AD and allergic rhinitis cases - 8,179 controls	Adults	Korean	3 novel SNPs associated with AD and allergic rhinitis in the Korean population: 1. 21q21.1, rs7275360, gene: NCAM2 2. 7q31.1, rs698195 3. 9q21.12, rs3750552, gene: FAM189A2	1. p=3.15×10^−6^ 2. p=3.72×10^−6^ 3. p=4.40×10^−6^ - NCAM2 - involved in neuronal differentiation - indirect link to allergic disease
Pasanen et al. (2024) (37) (genome-wide meta- analysis)	- 37,541 cases - 1,056,519 controls	Adults	European, North- American, Australian, Japanese	10 novel loci: 1. rs529979470, chromosome 2, gene: B3GNT7 2. rs11130215, chromosome 3, gene: BHLHE40 3. rs368546772, chromosome 3, gene: FOXP1 4. rs4580527, chromosome 3, genes: TRMT10C, SENP7 5. rs1172342321, chromosome 6, gene: KCNK5 6. rs2347784, chromosome 7, genes: KDELR2, DAGLB 7. rs182341709, chromosome 10, gene: LINC02661 8. rs933905994, chromosome 16, gene: IFT140 9. rs350143, chromosome 19, gene: ARID3A 10. rs465340, chromosome 21, genes: SAMSN1-AS1, SAMSN1	1. p=5.67×10^−9^ 2. p=8.99×10^−10^ 3. p=3.76×10^−10^ 4. p=8.35×10^−9^ 5. p=4.46×10^−8^ 6. p=8.76×10^−9^ 7. p=3.90×10^−8^ 8. p=3.48×10^−11^ 9. p=1.93×10^−8^ 10. p=3.14×10^−9^ - different variants in the FLG locus for early vs late-onset AD - larger effect estimates for tested variants in severe vs mild AD
Oliva et al. (2025) (38) (genome-wide meta- analysis)	- 56,146 cases - 602,280 controls	Adults	European, Asian, African	16 novel loci (chromosomes 1, 2, 5, 6, 7, 8, 10, 11, 12, 14, 17, 18; genes: UBXN11, BCL2L11, GLS, PPIL3, ANKRD55, ITK, IL22RA2, SCAF8, STEAP1B, CHD7, ANK3, GSTP1, WNK1, BATF, SOCS3, ALPK2)	- study enriched through fine-mapping, QTL colocalization, and cell-type enrichment

This table provides an overview of all the GWAS that have identified novel genetic loci associated with AD, according to the GWAS catalog, PubMed and PubMed Central. For each study, we have provided the first author, publication year, sample size, age group and ancestry, the key loci, SNPs and genes reported, and brief notes on their biological relevance. Only the GWAS reporting novel AD susceptibility loci were included. (AD, atopic dermatitis; GWAS, genome-wide association studies; IgE, immunoglobulin E; OR, odds ratio; QTL, quantitative trait loci; SNPs, single nucleotide polymorphisms).

The first GWAS focused on AD was conducted in 2009 by Esparza-Gordillo et al. (18), and it identified a relevant association between AD and a SNP localized on chromosome 11q13.5, near the C11orf30 gene. Furthermore, the study estimated that the homozygous status for rs7927894 (allele A) is present in approximately 13% in the European population, with the limitation that the study sample was exclusively composed of individuals of German descent. After a two-year gap, this GWAS was followed by a study on a Chinese Han cohort, conducted by Sun et al. (19), which identified two new susceptibility loci (5q22.1 and 20q13.33), encoding proteins involved in immune signaling in immune signaling (e.g., TNFRSF6B, ZGPAT) and mitochondrial metabolism (SLC25A46). Fueled by these novel findings, a series of follow-up and candidate gene studies were conducted on Chinese cohorts in the following years, aside from the GWAS that have subsequently emerged. A replication study conducted on a cohort from Singapore suggested a weak association at 20q13.33, raised the possibility that 5q22.1 associations may depend on environmental interactions, and has also indicated a strong association at the 10q21.2 locus (39), confirmed a few years later by Cai et al. (40). Gao et al. (41) have verified whether previously discovered allergic sensitization loci were also associated with AD in a Chinese cohort, suggesting shared genetic underpinnings between allergic sensitization and AD. Associations with AD have also been reported for UBASH3A gene polymorphisms (42), and within 11p13.5 (43), 2p13.3 (44), and 5q22.1 loci, the latter presenting insertion-deletion (indel) variants pointing to TMEM232 as a susceptibility gene (45). Jiang et al. (46) further strengthened this evidence by reporting associations at the 5q22.1 and 5q31 loci. These cumulative results and their broader significance were reinforced by a systematic review and meta-analysis conducted in 2023, which synthesized candidate-gene associations across European and Asian populations (47).

After the efforts of Esparza-Gordillo et al. (18) and Sun et al. (19), more GWAS have started to emerge, contributing with additional insights into the susceptibility to AD. Associations were identified at OVOL1, ACTL9, KIF3A (20), while further variants were reported at IL1RL1 and within the MHC (21). Additional loci pertaining to the epidermal differentiation complex (EDC), RAD50/IL13, MHC, and LRRC32 were identified, highlighting an overlap between the genetic basis of AD and psoriasis (22), while four novel loci were subsequently uncovered through dense genotyping with Immunochip (23). The number of established loci was expanded to 31 across multiple ancestries (24), with 14 of the previously established loci being confirmed in the same year, while also identifying two novel ones (XIRP2, DMRTA1) (25). The first Korean GWAS revealed 5 novel loci in a pediatric population with recalcitrant AD (26). More recently, in 2021, rare variant analyses detected 3 exonic variants in DUSP1, NOTCH4, and SLC9A4 (29), while trans-ethnic analyses highlighted Japanese-specific and shared loci (30). Further studies indicated the involvement of DSC1 and SERPINB7 in AD through novel pathways (31), progress within the EArly Genetics and Lifecourse Epidemiology (EAGLE) consortium has been summarized, confirming and extending previous findings (34), ancestry-specific loci have been identified in African Americans (35), and 10 novel loci have been pointed out through a comprehensive genome-wide meta-analysis at the end of 2024 (37). The most recent GWAS has integrated fine-mapping and Quantitative Trait Loci (QTL) colocalization, pinpointing more genes and cell types underlying AD pathophysiology (38). Together, these genome-wide efforts have now uncovered over 100 independent loci contributing to AD susceptibility and pathogenesis (details regarding all of the aforementioned GWAS can be found in **Table 1**).

However, not all GWAS pertaining to AD have focused exclusively on identifying novel susceptibility loci. Several studies have emphasized genetic overlap and comorbidity with related conditions, instead. Among the studies included in **Table 1**, Baurecht et al. (27) demonstrated a high degree of genomic coincidence between AD and psoriasis, with some loci exerting opposing risk effects, suggesting that shared biological mechanisms may act differently. Johansson et al. (28) have managed to identify 41 novel loci for asthma, hay fever, and/or eczema, 4 of which were uniquely associated with the combined phenotype, highlighting both disease-specific, and shared effects. Margaritte-Jeannin et al. (32) have reported OCA2 as a novel locus associated with asthma-plus-eczema comorbidity, linking the biological processes related to melanin synthesis to the skin barrier function. Shirai et al. (33) further demonstrated shared genetic components between allergic diseases (asthma, AD, AR) and autoimmune conditions, while Kim JW et al. (36) detected SNPs of suggestive significance for a combined AD-and-AR phenotype in a Korean cohort. In contrast, GWAS focusing on associations between AD and other comorbidities, without demonstrating novel AD loci as well, have yet to be included in **Table 1**. Such studies include Rosenberg et al. (48), who identified a novel locus for hand eczema and confirmed its genetic correlation with AD, and a very recent GWAS, published in June 2025 (not yet indexed on GWAS catalog (17)), that associated CACNA2D3 with multimorbidity of asthma and AD in children, but not with AD alone (49). Without studying the genome on such a broad level as GWAS, cross-trait analyses have also revealed broader pleiotropy for AD: shared loci have been identified between AD and bullous pemphigoid (50), as well as between AD and neuropsychiatric disorders (51). Together, these studies underscore the systemic and multidimensional nature of AD’s genetic architecture.

Apart from locus discovery, some GWAS-related efforts have focused on applying genetic insights in different contexts. For instance, Simard et al. (52) managed to develop a polygenic risk score in Canadian cohort, that was able to explain up to 37% of disease severity variance, demonstrating the predictive potential of known loci, while Stickley et al. (53) have taken completely different route, investigating gene-environment interactions that might shape the infant gut microbiota in asthma and atopy. These complementary approaches are meant to underscore how the genetic study of AD extends beyond locus discovery to encompass comorbidity, pleiotropy, and prediction.

A limitation of GWAS lies in the fact that they do not examine the entire genome, rather focusing on the direct analysis of the most common genetic variants. These studies subsequently rely on imputation techniques, which, although they offer the potential to infer rarer variants based on those already identified, are highly dependent on the size and quality of the reference panel used for imputation. Consequently, rare genetic variants are significantly less explored, even though they may exert moderate or even strong effects on complex phenotypic traits. A major step toward overcoming this limitation is the advancement of modern sequencing technologies which enable whole-genome sequencing, coupled with the continuous decrease in associated costs. It is anticipated that the cost of sequencing an entire human genome will decrease from approximately $600 to $100 in the near future (2).

## 3 Causal inference using Mendelian randomization in atopic dermatitis

The aforementioned GWAS have provided large amounts of data regarding AD, pinpointing associations with different pathways involved in etiopathogenesis or various comorbidities, identifying the genetic determinants of modifiable exposures and diverse phenotypic traits. However, these discoveries were only the starting point for uncovering causal relationships between AD and various factors, which can be further examined through Mendelian randomization (MR) analyses, as well as other approaches, such as randomized controlled trials and longitudinal cohort studies.

MR is a statistical method that takes advantage of the random allocation of alleles at conception, as described by Mendel’s first law (the law of segregation) and second law (the law of independent assortment). By using inherited germline genetic variants as instrumental variables, MR can draw causal inferences between different exposures and outcomes (54). Unlike traditional epidemiological studies, such as registry-based cohorts or population surveys, which could be limited by unmeasured confounding variables, MR leverages the fact that germline variants are established at conception and remain stable throughout life. This fact might overcome challenges by minimizing reverse causality, while also reducing the impact of many confounders (13, 15). MR can be applied on one or more population studies, through one-sample, two-sample or multivariable studies. The most commonly used in recent literature are the two-sample MR studies, in which the variables associated with the exposure are assessed in a different cohort than the outcome-derived variables, increasing statistical power compared to one-sample MR studies (55). For MR inferences to be valid, however, three key conditions must be met, namely: the genetic variant must be significantly associated with the exposure, the variant must be independent of confounding factors, and the exposure must be a direct consequence of that specific genetic variant (15).

**Figure 2:**
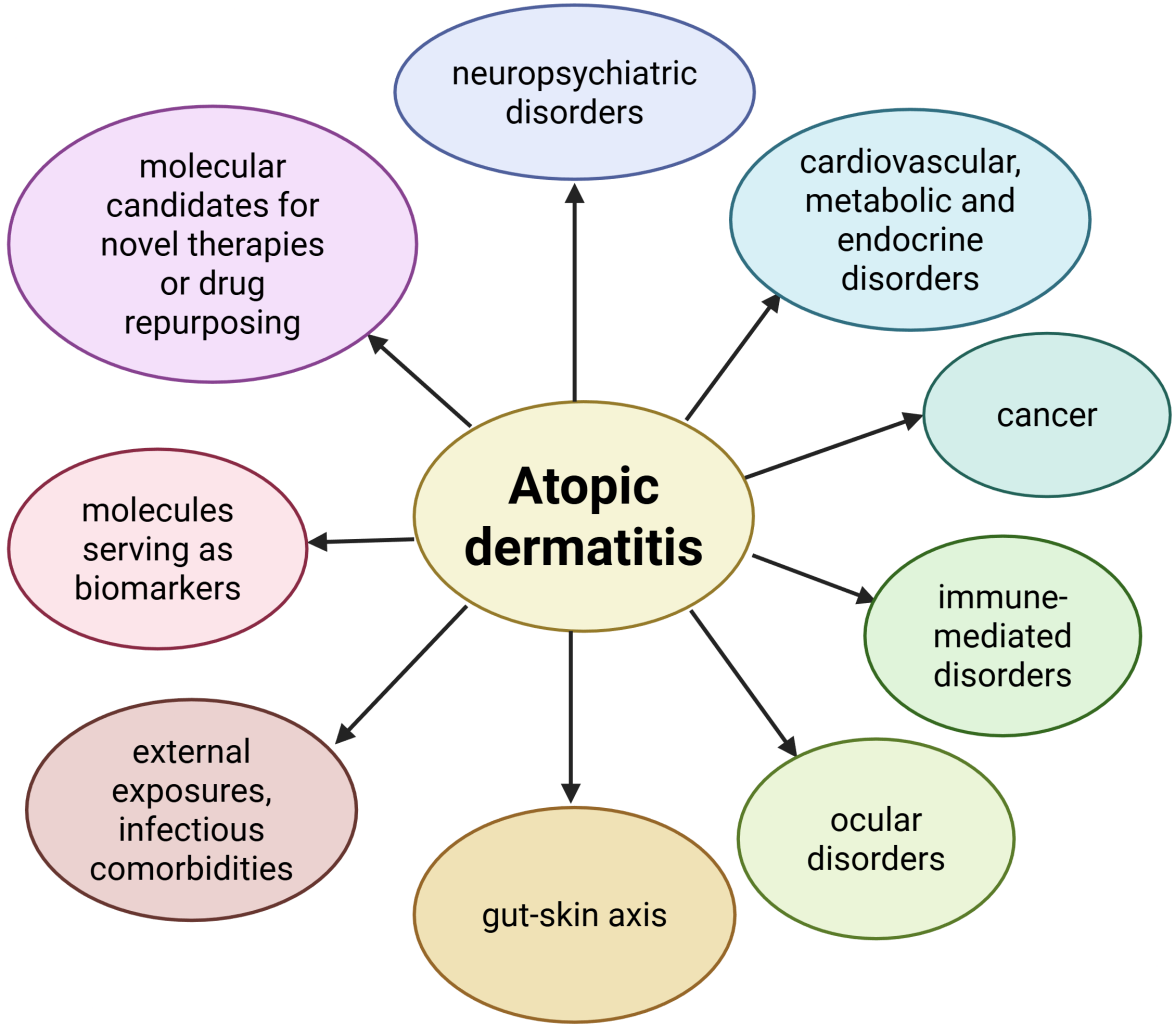
Areas in which causal inferences with AD have been identified through MR studies. Created in BioRender. Chera, A. (2026) https://BioRender.com/xpkw85t.

AD has recently started to be explored through the lens of MR. MR studies regarding AD have targeted a wide range of areas, assessing inferences with psychiatric and neurological conditions, cardiovascular and metabolic disorders, neoplasia, other immune-mediated conditions, ocular comorbidities, gut-skin axis, environmental exposures, infectious diseases, while also uncovering causal links with translational targets, which could highlight novel biomarkers, therapeutic targets, or even drug repurposing opportunities (**Figure 2**).

### 3.1 Causal associations between AD and psychiatric and neurological disorders

Associations between AD and psychiatric or neurological conditions have been hypothesized throughout time, therefore causal inference studies have become increasingly necessary. This fact has made room for many MR studies aiming to establish the causality between AD and different neuropsychiatric disorders, such as major depressive disorder (MDD), anxiety, bipolar disorder (BD), attention deficit hyperactivity disorder (ADHD), autism spectrum disorders (ASD), obsessive-compulsive disorder (OCD), and even Parkinson’s disease, migraines and brain neoplasia. **Table 2** (56–71) summarizes 16 relevant studies from 2021 to 2025 which illustrate causal links between AD and neuropsychiatric conditions.

**Table 2:** Published MR studies investigating causal associations between AD and psychiatric or neurological disorders.

Domain	Disease	First author (year)	Causal direction tested	*p*-value	Conclusion
Psychiatric entities	Major depressive disorder (MDD)	Cao et al. (2021) (56)	**1. MDD -> AD** **2. AD -> MDD**	**p_1_ = 1.76×10^−6^** **p_2_ = 7.57×10^−3^**	**Causal link (bidirectional)**
		Baurecht et al. (2021) (57)	AD -> MDD	p=0.466	No causal link
		Budu-Aggrey et al. (2021) (58)	AD -> MDD	p=0.50	No causal link
		Qi et al. (2022) (59)	**AD -> MDD**	**p=0.02**	**Causal link**
		Mo et al. (2024) (60)	1. AD -> MDD **2. MDD -> AD**	p_1_ = 0.18 **p**_2_ = **0.024**	**Causal link (unidirectional)**
		Cao et al. (2024) (61)	1. AD -> MDD 2. MDD -> AD	p_1_ = 0.17 p_2_ = 0.67	No causal link
		Wu et al. (2025) (62)	1. AD -> MDD 2. MDD -> AD	p_1_ = 0.53 p_2_ = 0.64	No causal link
		Fan et al. (2025) (63)	**1. AD -> MDD** 2. MDD -> AD	**p_1_ = 0.01** p_2_ = 0.77	**Causal link (unidirectional)**
	Anxiety	Baurecht et al. (2021) (57)	AD -> anxiety	p=0.18	No causal link
		Budu-Aggrey et al. (2021) (58)	AD -> anxiety	p=0.19	No causal link
		Cao et al. (2024) (61)	1. AD -> anxiety 2. anxiety-> AD	p_1_ = 0.10 p_2_ = 0.13	No causal link
		Wu et al. (2025) (62)	**1. AD -> anxiety** 2. anxiety-> AD	**p_1_ = 0.01** p_2_ = 0.46	**Causal link (unidirectional)**
	Bipolar disorder (BD)	Budu-Aggrey et al. (2021) (58)	AD -> BD	p=0.34	No causal link
		Mo et al. (2024) (60)	1. AD -> BD 2. BD -> AD	p_1_ = 0.89 p_2_ = 0.93	No causal link
		Cao et al. (2024) (61)	1. AD -> BD **2. BD -> AD**	p_1_ = 0.68 **p_2_ = 0.02**	**Causal link (unidirectional)**
		Wu et al. (2025) (62)	1. AD -> BD **2. BD -> AD**	p_1_ = 0.45 **p_2_ = 0.005**	**Causal link (unidirectional)**
		Fan et al. (2025) (63)	1. AD -> BD 2. BD -> AD	p_1_ = 0.98 p_2_ = 0.08	No causal link
	Neuroticism	Budu-Aggrey et al. (2021) (58)	AD -> neuroticism	p=0.64	No causal link
		Antonatos et al. (2024) (64)	1. AD -> worry **2. worry -> AD**	p_1_ = 0.27 **p_2_ = 3.97×10^−3^**	**Causal link (unidirectional)**
	Risk of suicide	Qi et al. (2022) (59)	AD -> suicidality	p=0.15	No causal link
		Wu et al. (2025) (62)	1. AD -> suicidality 2. suicidality -> AD	p_1_ = 0.19 p_2_ = 0.58	No causal link
	ADHD (Attention deficit hyperactivity disorder)	Ng et al. (2023) (65)	1. AD -> ADHD 2. ADHD -> AD	p_1_ = 0.70 p_2_ = 0.23	No causal link
		Cao et al. (2024) (61)	**1. AD -> ADHD** **2. ADHD -> AD**	**p_1_ = 0.03** **p_2_ = 9.2×10^−40^**	**Causal link (bidirectional)**
		Wu et al. (2025) (62)	1. AD -> ADHD 2. ADHD -> AD	p_1_ = 0.77 p_2_ = 0.43	No causal link
		Fan et al. (2025) (63)	1. AD -> ADHD 2. ADHD -> AD	p_1_ = 0.22 p_2_ = 0.41	No causal link
	Autism spectrum disorder (ASD)	Cao et al. (2024) (61)	**1. AD -> ASD** 2. ASD -> AD	**p_1_ = 0.01** p_2_ = 0.20	**Causal link (unidirectional)**
		Wu et al. (2025) (62)	1. AD -> ASD 2. ASD -> AD	p_1_ = 0.19 p_2_ = 0.68	No causal link
		Fan et al. (2025) (63)	1. AD -> ASD 2. ASD -> AD	p_1_ = 0.11 p_2_ = 0.07	No causal link
	Anorexia nervosa (AN)	Cao et al. (2024) (61)	1. AD -> AN **2. AN -> AD**	p_1_ = 0.09 **p_2_ = 4.4×10^−10^**	**Causal link (unidirectional)**
	Tic disorders (including Tourette syndrome)	Cao et al. (2024) (61)	1.AD->Tourette syndrome 2.Tourette syndrome-> AD	p_1_ = 0.95 p_2_ = 0.69	No causal link
		Fan et al. (2025) (63)	1. AD -> tic disorder 2. tic disorder -> AD	p_1_ = 0.07 p_2_ = 0.38	No causal link
	Obsessive- compulsive disorder (OCD)	Wu et al. (2025) (62)	1. AD -> OCD 2. OCD -> AD	p_1_ = 0.91 p_2_ = 0.87	No causal link
		Fan et al. (2025) (63)	1. AD -> OCD 2. OCD -> AD	p_1_ = 0.21 p_2_ = 0.76	No causal link
	Schizophrenia	Mo et al. (2024) (60)	1. AD -> schizophrenia 2. schizophrenia -> AD	p_1_ = 0.40 p_2_ = 0.88	No causal link
		Cao et al. (2024) (61)	1. AD -> schizophrenia 2. schizophrenia -> AD	p_1_ = 0.07 p_2_ = 0.22	No causal link
		Wu et al. (2025) (62)	1. AD -> schizophrenia 2. schizophrenia -> AD	p_1_ = 0.50 p_2_ = 0.89	No causal link
		Fan et al. (2025) (63)	1. AD -> schizophrenia 2. schizophrenia -> AD	p_1_ = 0.31 p_2_ = 0.55	No causal link
Neurological diseases	Parkinson’s disease (PD)	Zhou et al. (2024) (66)	**1. AD -> PD** 2. PD -> AD	**p_1_ = 0.02** p_2_ = 0.41	**Causal link (unidirectional)** **-** AD=protective factor against PD
	Brain cancer	Xin et al. (2024) (67)	**AD -> brain cancer**	**p=9.6×10^−3^**	**Causal link**
	Dementia	Gwak et al. (2024) (68)	AD -> dementia (any)	p=0.90	No causal link
	Insomnia	Ni et al. (2025) (69)	**AD -> insomnia**	**p=0.03**	**Causal link**
	Migraines	Li et al. (2024) (70)	**1. AD -> migraines** 2. migraines -> AD	**p_1_ = 0.04** p_2_ = 0.60	**Causal link (unidirectional)**
	Menière disease	Qin et al. (2024) (71)	**AD -> Menière disease**	**p=0.029**	**Causal link**

AD, atopic dermatitis; ADHD, attention deficit hyperactivity disorder; AN, anorexia nervosa; ASD, autism spectrum disorder; BD, bipolar disorder; MDD, major depressive disorder; OCD, obsessive-compulsive disorder; PD, Parkinson’s disease.

The bold formatting is relevant for highlighting the causal links between AD and different comorbidities, according to the p-values (the causal directions that were linked to p-values lower than 0.05 were formatted in bold for a better visualisation).

MR evidence assessing the causal relationship between AD and psychiatric or neurological conditions paints a heterogeneous picture (**Table 2**). For major depressive disorder, the conclusions are equally split; some studies reported causal associations, either unidirectional - two studies indicated that AD might increase the risk of developing MDD (59, 63), while Mo et al. have found the reversed causality relationship to be statistically significant (60) -, or bidirectional (56), while four studies found no evidence of causality between these two entities (57, 58, 61, 62). For anxiety, most analyses indicated no causal link, though Wu et al. (62) suggested a significant unidirectional effect from AD to anxiety. In the case of bipolar disorder, findings were also mixed: two studies suggested a reverse causal association from bipolar disorder to AD (61, 62), while three articles reported no causal links (58, 60, 63). Evidence for risk of suicide, tic disorders, OCD and schizophrenia is consistently negative, with no causal relationships detected across studies (59–63). For ADHD and ASD, the results were divergent: one article supported causal bidirectional associations between AD and ADHD and a unidirectional link between AD and ASD (61), while other large-scale studies found no evidence (62, 63, 65). Anorexia nervosa showed some evidence of reverse causality toward AD, but not the opposite (61), while for neuroticism, Antonatos et al. (64) reported a unidirectional association from worry to AD. Regarding neurological conditions, the evidence is less nuanced. AD appears to act as a protective factor against Parkinson’s disease (66), and as a risk factor for brain cancer (67), insomnia (69), migraines (70), and Menière’s disease (71), without any causal association with dementia (including Alzheimer’s disease, or vascular dementia (68)).

While AD shows potential causal links with select psychiatric and neurological disorders, particularly MDD, anxiety, bipolar disorder, and certain neurological outcomes, much of the evidence remains inconsistent across studies. These findings underscore the complexity of shared genetic and environmental pathways, suggesting that comorbidities between AD and mental or neurological conditions may reflect many joined causal mechanisms, pleiotropy, and confounding factors, rather than a uniform causal pathway.

### 3.2 Causal associations between AD and cardiovascular, metabolic or endocrine disorders

Earlier observational and epidemiological studies conducted by researchers, such as Standl et al. (72), suggested modest associations of atopic dermatitis with angina pectoris, arterial hypertension, and peripheral arterial disease, but found no robust genetic overlap with cardiovascular disease. Even though these observations were valuable, they also exemplify the limitations of traditional epidemiological designs in disentangling causality, thereby underscoring the need for conducting MR studies in order to clarify whether these associations reflect true causal relationships. **Table 3** (73–87) summarizes the most important findings from 15 MR studies which address potential cusal links between AD and various cardiovascular, metabolic and endocrine disorders and risk factors.

**Table 3:** Published MR studies investigating causal associations between AD and cardiovascular, metabolic or endocrine disorders/risk factors.

Disease/risk factor	First author (year)	Causal direction tested	*p*-value	Conclusion
Heart failure (HF)	Chen H et al. (2022) (73)	**AD -> HF**	**p=1.11×10^−4^**	**Causal link**
	Qi et al. (2022) (74)	AD -> HF	p=0.11	No causal link
	Guo et al. (2022) (75)	**AD -> HF**	**p=0.01**	**Causal link**
Coronary artery disease (CAD)	Chen H et al. (2022) (73)	AD -> CAD	p=0.88	No causal link
	Huang et al. (2022) (76)	AD -> CAD	p=0.90	No causal link
	Qi et al. (2022) (74)	AD -> CAD	p=0.98	No causal link
Myocardial infarction (MI)	Chen H et al. (2022) (73)	AD -> MI	p=0.75	No causal link
	Huang et al. (2022) (76)	AD -> MI	p=0.18	No causal link
	Qi et al. (2022) (74)	AD -> MI	p=0.32	No causal link
Angina pectoris	Qi et al. (2022) (74)	AD -> angina pectoris	p=0.36	No causal link
Atrial fibrillation (AF)	Chen H et al. (2022) (73)	AD -> AF	p=0.78	No causal link
Stroke (any subtype)	Chen H et al. (2022) (73)	AD -> stroke	p=0.54	No causal link
	Huang et al. (2022) (76)	AD -> ischemic stroke	p=0.85	No causal link
	Qi et al. (2022) (74)	AD -> stroke	p=0.36	No causal link
Chronic kidney disease (CKD) (cardiometabolic outcome)	Zhang et al. (2023) (77)	**1. AD -> CKD** **2. CKD -> AD**	**p_1_ = 0.013** **p_2_ = 0.019**	**Causal link (bidirectional)**
Erectile dysfunction (ED)	Yu et al. (2024) (78)	**AD -> ED**	**p=0.01**	**Causal link** - AD=protective factor against ED
Obesity/Body-mass index (BMI)	Yew et al. (2020) (79)	**1. high BMI -> AD** 2. AD -> high BMI	**p_1_ = 0.01** p_2_ = 0.86	**Causal link (unidirectional)**
	Budu-Aggrey et al. (2020) (80)	**1. high BMI -> AD** 2. AD -> high BMI	**p_1_ = 0.02** p_2_ = 0.24	**Causal link (unidirectional)**
	Li Y et al. (2023) (81)	**1. obesity -> AD** 2. AD -> obesity	**p_1_ = 0.002** p_2_ = 0.60	**Causal link (unidirectional)**
	Li Z et al. (2024) (82)	**high BMI -> AD**	**p=0.022**	**Causal link**
Diabetes mellitus (DM)	Chen Y et al. (2022) (83)	**diabetes age of onset -> AD**	**p=2.77×10^−5^**	**Causal link**
	Lu et al. (2023) (84)	**1. AD -> type 1 DM** **2. AD -> type 2 DM**	**p_1_ = 0.006** **p_2_ = 0.003**	**Causal link (bidirectional)**
Thyroid dysfunction (thyroidectomy, hyper- and hypothyroidism)	You et al. (2024) (85)	1. AD -> thyroidectomy **2. thyroidectomy -> AD** 3. AD -> hypothyroidism 4. hypothyroidism -> AD 5. AD -> hyperthyroidism 6. hyperthyroidism -> AD	p_1_ = 0.86 **p_2_ = 0.001** p_3_ = 0.81 p_4_ = 0.09 p_5_ = 0.25 p_6_ = 0.92	**Causal link** - thyroidectomy= protective factor against AD
	Yin et al. (2025) (86)	**1. AD -> hypothyroidism** 2. AD -> hyperthyroidism	**p_1_ = 0.006** p_2_ = 0.08	**Causal link** - AD=risk factor for hypothyroidism
Sarcopenia (linked to metabolic dysfunction)	Tang et al. (2024) (87)	**AD -> appendicular lean mass**	**p=0.003**	**Causal link**

AD, atopic dermatitis; AF, atrial fibrillation; BMI, body-mass index; CAD, coronary artery disease; CKD, chronic kidney disease; DM, diabetes mellitus; ED, erectile dysfunction; HF, heart failure; MI, myocardial infarction.

The bold formatting is relevant for highlighting the causal links between AD and different comorbidities, according to the p-values (the causal directions that were linked to p-values lower than 0.05 were formatted in bold for a better visualisation).

Several MR studies indicate that AD is not causally linked to coronary artery disease, myocardial infarction, atrial fibrillation, angina, or stroke (73, 74, 76), however, causality between AD and heart failure has been identified in two studies (73, 75). Four MR studies consistently showed that higher BMI and obesity increase the risk of AD, while reverse causation was not supported (79–82). Pertaining to endocrinologic diseases and imbalances affecting metabolism, causal associations with AD have revolved around diabetes mellitus and thyroid dysfunctions: Chen Y. et al. (83) demonstrated that earlier onset of diabetes increases the risk of AD, Lu et al. (84) showed that AD itself is able to increase the risk of both type 1 and type 2 diabetes, while You et al. (85) suggested thyroidectomy may be protective against AD, and Yin et al. (86) found AD to be a risk factor for hypothyroidism. Other unexpected exposures and outcomes have also been explored in regards to AD, some studies indicating protective effects of AD against erectile dysfunction (78), bidirectional causal links with chronic kidney disease (77), and the contribution of AD in reducing appendicular lean mass, linking it with sarcopenia (87).

Even though AD does not appear to have strong causal associations with major cardiovascular entities, such as myocardial infarction or stroke, it does play a role in metabolic dysregulation, particularly in relation to obesity, diabetes, chronic kidney disease, thyroid function, and sarcopenia.

### 3.3 Causal associations between AD and neoplasia

Throughout the years, MR studies have also explored causal inferences between AD and cancer. The most important data from 10 such original articles has been summarized in **Table 4** (88–97).

**Table 4:** Published MR studies investigating causal associations between AD and different types of cancer.

Cancer localization	First author (year)	Causal direction tested	p-value	Conclusion
Brain	Disney-Hogg et al. (2018) (88)	**AD -> glioma**	**p=0.041**	**Causal link**
Esophagus	Liu et al. (2024) (89)	**AD -> esophageal cancer**	**p=0.011**	**Causal link** - AD= protective factor against esophageal cancer - only found in a Japanese cohort
Stomach	Wei et al. (2023) (90)	AD -> gastric cancer	p=0.33	No causal link
Colon and rectum	Zhan et al. (2024) (91)	**AD -> colorectal cancer (CRC)**	**p=0.002**	**Causal link** - AD= protective factor against CRC
	Alduhayh et al. (2025) (92)	**AD or AR -> CRC (overall)** **AD or AR -> early onset CRC**	**p_1_ = 0.002** **p_2_ = 2.16×10^−5^**	**Causal link** - AD= protective factor against overall and early-onset CRC
Breast	Jiang et al. (2020) (93)	AD -> breast cancer	p=0.95	No causal link
	Yang et al. (2024) (94)	1. AD -> breast cancer **2. breast cancer -> AD**	p_1_ = 0.79 **p_2_ = 0.047**	**Causal link (unidirectional)** - breast cancer=protective factor against AD
Prostate	Jiang et al. (2020) (93)	AD -> prostate cancer	p=0.93	No causal link
Lung	Huang et al. (2024) (95)	**AD -> lung adenocarcinoma (LUAD)**	**p=0.007**	**Causal link** - AD= protective factor against LUAD
Lymphoma	O’Hagan et al. (2023) (96)	**AD -> mature T/NK-cell lymphomas**	**p=0.007**	**Causal link** - only found in a Finnish cohort
	Song et al. (2025) (97)	**AD -> T/NK-cell lymphoma** **AD -> mantle cell lymphoma**	**p_1_ = 0.001** **p_2_ = 4.25×10^−3^**	**Causal links** - AD=protective factor for lymphoma

AD, atopic dermatitis; AR, allergic rhinitis; CRC, colorectal cancer; LUAD, lung adenocarcinoma.

The bold formatting is relevant for highlighting the causal links between AD and different comorbidities, according to the p-values (the causal directions that were linked to p-values lower than 0.05 were formatted in bold for a better visualisation).

Broader multi-cancer analyses have also been conducted, but have lacked any significant causal associations. For example, in 2023, Liu et al. (98) have conducted a study pertaining to AD, searching for causal links to many different types of cancer (colorectal, lung, skin, head and neck, bladder, prostate, breast, ovarian, endometrial, leukemia, lymphoma and pan-cancers), but have not found any significant causality (all p-values were >0.05). While these findings were supported by some of the more targeted studies that have been conducted (e.g. no causal links have been identified between AD and gastric (90) or prostate (93) cancers), AD has been pointed as a significant pawn in causality by some studies regarding glioma, esophageal, colorectal, breast, lung cancers and lymphoma. More specifically, protective values have been attributed to AD in regards to esophageal cancer in a Japanese cohort (89), overall and early-onset colorectal cancer (91, 92), lung adenocarcinoma (95) and T/NK-cell and mantle lymphoma (96, 97). Interestingly, Yang et al. (2024) (94) have found evidence for reversed causality, linking breast cancer to protective properties against AD.

To conclude, causal inferences have been found between AD and neoplasia in different sites indicating potential protective roles of AD, probably linked to shared immune pathways and other mechanisms. However, further research targeting larger, multi-ancestry cohorts is needed to confirm these findings and resolve inconsistencies across studies.

### 3.4 Causal associations between AD and other immune-mediated disorders

Recent MR studies have systematically investigated whether AD shares causal relationships with other immune-mediated diseases, in order to clarify the extent of comorbidity beyond observational overlaps. **Table 5** (99–103) shows the most important findings from 17 MR studies targeting causality between AD and immune-related afflictions, pertaining to many medical specialties, such as dermatology, gastroenterology, rheumatology, nephrology, or oto-rhino-laringology.

**Table 5:** Published MR studies investigating causal associations between AD and different types of immune-mediated diseases.

Domain	Disease	First author (year)	Causal direction tested	*p*-value	Conclusion
Dermatology	Psoriasis	Zhao et al. (2024) (99)	**1. AD -> psoriasis** **2. psoriasis -> AD**	**p_1_ = 0.01** **p_2_ = 0.001**	**Causal link (bidirectional)**
	Alopecia areata (AA)	O’Hagan et al. (2023) (100)	**1. AD -> AA** 2. AA -> AD	**p_1_ = 0.01** p_2_ = 0.73	**Causal link (unidirectional)**
		Zhou et al. (2023) (101)	**AD -> AA**	**p=0.006**	**Causal link**
	Hidradenitis suppurativa (HS)	Tang et al. (2023) (102)	**1. AD -> HS** 2. HS -> AD	**p_1_ = 0.04** p_2_ = 0.825	**Causal link (unidirectional)**
	Vitiligo	Zhou et al. (2023) (101)	AD -> vitiligo	p=0.54	No causal link
		Chen C et al. (2024) (103)	AD -> vitiligo	p=0.32	No causal link
	Bullous pemphigoid (BP)	Chen C et al. (2024) (103)	AD -> BP	p=0.64	No causal link
Gastro- enterology	Inflammatory bowel disease (IBD) - Crohn’s disease (CD), ulcerative colitis (UC)	Gu et al. (2022) (104)	**1. AD -> IBD** 2. IBD -> AD	**p_1_ = 0.43** p_2_ = 0.01	**Causal link (unidirectional)**
		Meisinger et al. (2022) (105)	1. AD -> CD **2. CD -> AD** **3. AD -> UC** 4. UC -> AD	p_1_ = 0.07 **p_2_ = 0.02** **p_3_ = 0.02** p_4_ = 0.83	**Causal links (unidirectional)**
		Wang et al. (2022) (106)	1. AD -> CD 2. CD -> AD 3. AD -> UC 4. UC -> AD	**p_1_<0.001** **p_2_<0.001** p_3_ = 0.12 **p_4_ = 0.015**	**Causal links (bidirectional for CD, unidirectional for UC)**
		Zheng et al. (2024) (107)	1. AD -> CD 2. CD -> AD 3. AD -> UC 4. UC -> AD	**p_1_ = 0.02** p_2_ = 0.36 **p_3_ = 0.06** p_4_ = 0.29	**Causal links (unidirectional)**
		Chen C et al. (2024) (103)	1. AD -> CD 2. AD -> UC	**p_1_<0.001** **p_2_ = 0.002**	**Causal links (unidirectional)**
	Celiac disease	Ge et al. (2025) (108)	**celiac disease->AD**	**p=5.75×10^-4^**	**Causal link**
		Wang et al. (2025) (109)	**AD-> celiac disease**	**p<0.001**	**Causal link**
Rheumatology	Rheumatoid arthritis (RA)	Zhou et al. (2023) (101)	**AD -> RA**	**p=0.007**	**Causal link**
		Chen et al. (2023) (110)	**1. AD -> RA** 2. RA -> AD	**p_1_ = 0.019** p_2_ = 0.34	**Causal link (unidirectional)**
		Chen C et al. (2024) (103)	AD -> RA	p=0.46	No causal link
		Zhao et al. (2024) (111)	IL-6 receptor inhibitors (used for treating RA) -> AD	**p=6.5×10^-4^**	**Causal link** **(AD might be triggered as an adverse reaction)**
	Systemic lupus erythematosus (SLE)	Zhou et al. (2023) (101)	AD -> SLE	p=0.25	No causal link
		Chen C et al. (2024) (103)	AD -> SLE	p=0.40	No causal link
		Xia et al. (2024) (112)	**SLE -> AD**	**p=0.03**	**Causal link**
	Psoriatic arthritis (PSA)	Zhao et al. (2024) (99)	**1. AD -> PSA** 2. PSA -> AD	**p_1_ = 0.03** p_2_ = 0.21	**Causal link (unidirectional)**
	Ankylosing spondylitis (AS)	Zhou et al. (2023) (101)	AD -> AS	p=0.19	No causal link
		Chen C et al. (2024) (103)	AD -> AS	p=0.12	No causal link
Nephrology	IgA nephropathy	Li et al. (2024) (113)	**AD -> IgA nephropathy**	**p=0.04**	**Causal link**
		Cao et al. (2024) (114)	**AD -> IgA nephropathy**	**p=0.007**	**Causal link**
	Interstitial cystitis (IC)	Wu et al. (2025) (115)	AD -> IC	p=0.55	No causal link
Oto-rhino- laryngology	Chronic adednotonsillar diseases (CATD)	Chen H et al. (2024) (116)	**AD -> CATD**	**p=0.006**	**Causal link**
Neurology	Multiple sclerosis (MS)	Chen C et al. (2024) (103)	AD -> MS	p=0.96	No causal link

AA, alopecia areata; AD, atopic dermatitis; AS, ankylosing spondylitis; BP, bullous pemphigoid; CATD, chronic adednotonsillar diseases; CD, Crohn’s disease; IBD, inflammatory bowel disease; IC, interstitial cystitis; HS, hidradenitis suppurativa; MS, multiple sclerosis; PSA, psoriatic arthritis; RA, rheumatoid arthritis; SLE, systemic lupus erythematosus; UC, ulcerative colitis.

The bold formatting is relevant for highlighting the causal links between AD and different comorbidities, according to the p-values (the causal directions that were linked to p-values lower than 0.05 were formatted in bold for a better visualisation).

In dermatology, robust bidirectional causal links have been established between AD and psoriasis (99), alongside unidirectional associations with alopecia areata (100, 101) and hidradenitis suppurativa (102). In contrast, no convincing causal evidence has been found for vitiligo or bullous pemphigoid (103). From a gastroenterologic point of view, all MR studies pertaining to gastrointestinal immune-mediated afflictions have demonstrated significant causal links to AD, suggesting intricate etiopathogenic pathways. Some support a causal role of AD in inflammatory bowel disease, with subtype-specific effects indicating bidirectional associations with Crohn’s disease and unidirectional links with ulcerative colitis (103–107), while bidirectional causality has also been demonstrated between AD and celiac disease, with recent evidence suggesting AD as a risk factor for celiac disease, as well as celiac disease increasing the risk of developing AD (108, 109). Regarding rheumatological diseases, AD has been shown to increase the risk of rheumatoid arthritis (101, 110), though this effect was not consistently replicated across all studies (103), while IL-6 receptor inhibitors, which are used for treating this pathological entity, have been linked to increased risk of triggering AD as an adverse event, and they should be administered with caution in patients with clinical history of AD (111). In regards to systemic lupus erythematosus, there was only one study proving that it can increase the risk of AD (112). A unidirectional causal link has been identified between AD and psoriatic arthritis (99), while no associations have been observed for ankylosing spondylitis (101, 103). Beyond these domains, AD has been associated with an increased risk of IgA nephropathy (113, 114) and chronic adenotonsillar disease (116), whereas no consistent associations were identified between AD and multiple sclerosis (103) or interstitial cystitis (115).

MR studies reveal AD as a ‘systemic’ immune-mediated disorder, with shared causal pathways across dermatologic, gastrointestinal, rheumatologic, and renal diseases. These findings carry important clinical implications, supporting the need for careful screening of AD patients for comorbid immune-mediated diseases, anticipating treatment-related interactions, and guiding precision medicine approaches in complex immunologic conditions.

### 3.5 Causal associations between AD and ocular disorders

MR studies have also explored the potential causal relationships between atopic AD and a spectrum of ocular diseases. The findings so far point to selective associations: while most analyses did not support a causal link between AD and keratoconus (117, 119, 120), one study identified a unidirectional effect of keratoconus increasing AD risk (118). In contrast, AD showed significant causal effects on senile cataract (121), conjunctivitis and keratitis (120), whereas no causal associations were observed with myopia (122), hordeolum, or pterygium (120). All this information has been summarized in **Table 6** (117–120).

**Table 6:** Published MR studies investigating causal associations between AD and ocular diseases*. (AD, atopic dermatitis)*.

Disease	First author (year)	Causal direction tested	*P*-value	Conclusion
Keratoconus	Xu et al. (2024) (117)	AD -> keratoconus	p=0.21	No causal link
	Chang et al. (2024) (118)	1. AD -> keratoconus **2. keratoconus -> AD**	p_1_ = 0.71 **p_2_ = 4.16×10^−4^**	**Causal link (unidirectional)**
	Yang et al. (2025) (119)	AD -> keratoconus	p=0.08	No causal link
	Zhou et al. (2023) (120)	AD -> keratoconus	p=0.23	No causal link
Senile cataract	Yuan et al. (2024) (121)	**AD -> senile cataract**	**p=0.04**	**Causal link**
Conjunctivitis	Zhou et al. (2023) (120)	**AD->conjunctivitis (all types)**	**p=8.65×10^-13^**	**Causal link**
Myopia	Wei et al. (2025) (122)	AD -> myopia	p=0.31	No causal link
Keratitis	Zhou et al. (2023) (120)	**AD -> keratitis**	**p=0.04**	**Causal link**
Hordeolum	Zhou et al. (2023) (120)	AD -> hordeolum	p=0.27	No causal link
Pterygium	Zhou et al. (2023) (120)	AD -> pterygium	p=0.79	No causal link

These findings support integrating ocular symptoms or comorbidities as part of systemic, associated manifestations of AD, supporting early ophthalmologic screening and preventive strategies in affected patients.

### 3.6 Causal associations between AD and the gut-skin axis

Healthy skin is able to integrate its function as a barrier for external pathogens with its property of sustaining a veritable ecosystem, capable of hosting diverse microbial communities. Dysbiosis of the skin and gut microbiota has been repeatedly observed in AD, though the direction of the causal relationship has remained unclear. MR has emerged as a valuable tool for clarifying whether dysbiosis is a cause or a consequence of AD, offering responses by integrating GWAS data with microbial composition, minimizing confounding factors and reverse causation. Recent MR analyses have revealed significant causal relationships between microbial taxa and inflammatory skin diseases, including AD, underscoring the importance of the interactions between the host and the microbiome within disease pathogenesis (173). Some microbial taxa exert protective effects against AD, while others seem to increase disease risk, highlighting a complex balance of microbial influence in disease susceptibility. For instance, taxa such as Bifidobacterium and Christensenellaceae have shown protective associations, whereas Bacteroidaceae, and Prevotella were implicated as risk factors (123–127). Similar findings extend to the skin microbiome, where certain microbial variants were linked to either increased risk or protection (130, 131). For detailed associations across microbial lineages, see **Table 7** (123–131).

**Table 7:** Members of the gut or skin microbiota which have been causally associated with AD through MR studies, either as protective or risk factors.

Microbial taxa/lineages with protective effects against AD	Microbial taxa/lineages increasing the risk of AD
Phyl. Tenericutes (123) - gut	Fam. Clostridiaceae (123) - gut
Cl. Mollicutes (123, 124) - gut	Fam. Bacteroidaceae, Gen. Bacteroides (123, 124) - gut
Cl. Clostridia, Or. Clostridiales (123, 124) - gut	Gen. Anaerotruncus (123) - gut
Fam. Bifidobacteriaceae, Or. Bifidobacteriales, Gen. Bifidobacterium (123, 124) - gut	Gen. Lachnospiraceae (123, 124) - gut
Gen. Christensenellaceae (123–125) - gut	Gen. Prevotella, Fam. Prevotellaceae (126, 127) - gut
Gen. Intestinimonas (124) - gut	Gen. Sellimonas (123) - gut
Gen. Eubacterium brachy group (124, 125), Gen. Eubacterium coprostanoligenes group (128) - gut	Gen. Ruminococcaceae NK4A214 group, Gen. Ruminococcaceae UCG011 (125, 128) - gut
Fam. Veillonellaceae (127) - gut	Or. Pasteurellales, Fam. Pasteurellaceae (129) - gut
Gen. Ruminococcaceae UCG004 (127), Gen. Ruminococcaceae UCG003 (128) - gut	Fam. Burkholderiales, Sp. Burkholderiales bacterium_1_1_47 (129) - gut
Or. Desulfovibrionales (123) - gut	Sp. Desulfovibrio piger (129) - gut
Phyl. Actinobacteria (123) - gut	***Gen. Dialister*** (126) **- *gut - contradictory effects***
Or. Actinomycetales (129) - gut	***Gen. Family XIII UCG001*** (128) **- *gut - contradictory effects***
Fam. Micrococcaceae (129) - gut	ASV006 (130) - skin, dry microenvironment
Fam. Oscillospiraceae, Gen. Oscillibacter (129) - gut	ASV076 (130) - skin, dry microenvironment
Gen. Rothia, Sp. Rothia mucilaginosa (129) - gut	ASV100 (131) - skin, dry microenvironment
Gen. Collinsella (129) - gut	
Gen. Pseudoflavonifractor (129) - gut	
Sp. Roseburia hominis (129) - gut	
Sp. Parabacteroides merdae (129) - gut	
***Gen. Dialister*** (124, 125, 127, 128) **- *gut - contradictory effects***	
***Gen. Family XIII UCG001*** (127) **- *gut - contradictory effects***	
Gen. Kocuria (130) - skin, dry microenvironment	
Gen. Enhydrobacter (131) - skin, moist microenvironment	
Fam. Streptococcaceae (131) - skin, dry microenvironment	
Cl. Bacilli (131) - skin, sebaceous microenvironment	
Gen. Corynebacterium (131) - skin, dry microenvironment	
ASV063 (131) - skin, sebaceous microenvironment	

AD, atopic dermatitis; Cl., class; Fam., family; Gen., genus; Or., order; Phyl, phylum; Sp., species.

The entries with contradictory evidence were formatted in italic; since there was evidence for those entries as both protective and risk factors for AD, they are present in both columns.

To summarize, MR studies support a causal role of the microbiome in AD, with different microbial taxa conferring either risk or protection. These findings underscore the potential of microbiome-targeted interventions, which include probiotics, prebiotics, or microbiota-directed therapies, as adjuvants in AD prevention and management. However, future studies will be needed in order to refine these associations and translate them into clinical practice, as a promising step toward precision medicine.

### 3.7 Causal associations between AD and external exposures or infectious comorbidities

Lifestyle, environmental, and infectious exposures are able to exert important influences on AD, and MR studies have come to aid in clarifying the exact causal inferences with specific external exposures, as shown in **Table 8** (132–144), which comprises the most important findings from 13 such studies.

**Table 8:** Published MR studies investigating causal associations between AD and different types of exposures (dietary, behavioural, environmental, infectious).

Type of exposure	Specific agent	Causal link with AD
**Dietary**	Meat	beef intake - protective factor against AD (132)
	Wheat products	Contradictory evidence: - white bread intake - protective factor against AD (132) - never eating wheat products - protective factor against AD (133)
	Tea intake	protective factor against AD (133–135)
**Behavioural**	Smoking	risk factor for AD (136)
		maternal smoking around birth - risk factor for child AD (137)
	Alcohol (prenatal exposure)	prenatal exposure - no effect on child AD (138)
**Environmental**	Air pollution	PM10 (particulate matter, Φ ≤10μm) - risk factor for AD (139)
**Biological - infectious**	SARS-CoV-2	common inflammatory pathways, but no effect on AD (140, 141)
	Influenza	common inflammatory pathways, but no effect on AD (142)
	Impetigo	AD - risk factor for developing impetigo (143)
	Bronchiectasis (potential consequence of respiratory infections)	AD - risk factor for developing bronchiectasis (144)

AD, atopic dermatitis.

Within dietary and fluid-intake factors, the most compelling evidence has been linked to the protective effects of tea consumption against AD (133–135). Smoking emerged as a clear risk factor for AD, with both personal smoking and maternal smoking around birth increasing susceptibility of developing AD (136, 137). Prenatal alcohol exposure, however, did not show a causal influence on childhood AD (138). From an environmental perspective, air pollution, particularly exposure to particulate matter of less than 10 μm in diameter (PM10), was identified as a clear risk factor for AD (139). Pertaining to biological agents that could be able to influence AD, it seems that viral agents are less inclined to influence, or be influenced, in the context of AD. Specifically, SARS-CoV-2 and influenza did not have any causal links to AD (140–142), while reversed causal inferences were detected for Staphylococcus aureus skin infections, AD becoming a risk factor in this context (143).

To sum up, MR studies have linked modifiable exposures, such as diet, smoking, pollution, to protective or detrimental properties in regards to AD, whereas AD itself predisposes to infectious diseases or complications, such as impetigo and bronchiectasis. These insights have clear clinical implications, supporting lifestyle interventions and environmental health policies in AD prevention, while emphasizing the need for vigilance in managing infection risk in affected patients.

### 3.8 Mendelian randomization in AD biomarker discovery

Reliable biomarker identification is crucial for understanding and improving the management of AD. Conventional, or classic biomarkers, such as blood cells and micronutrients, have long been investigated, occupying a central role in monitoring diseases. However, MR studies are able to provide more robust causal inference, offering valuable insights in biomarker discovery especially when coupled with multi-omic approaches, integrating proteomics, metabolomics, transcriptomics, lipidomics, and immunomics data for uncovering molecules which serve as novel, so-called ‘omics biomarkers’. Both the conventional and the novel approaches in identifying biomarkers for AD have been summarized in **Table 9** (145–167).

**Table 9:** Summary of the most important findings regarding causal inferences between AD and different types of biomarkers, either traditional/classic serum biomarkers, or novel ones, identified through omics studies.

>Biomarker category	Specific markers	Correlations to AD
Conventional biomarkers	Hemogram	- increased eosinophils and basophils -> potential risk factors for AD (145) - low lymphocyte count -> potential risk factor for AD (145)
	Zinc, Selenium	- Zn, Se -> potential protective factors against AD (146)
	Vitamin D	- no effects on AD (147) - increased vitamin D levels -> AD (148) (risk factor) - AD -> increased vitamin D levels (149)
	Other vitamins	- vitamin E -> AD (protective factor) (150, 151) - no causal links between AD and vitamin C, carotene (150) - vitamin A -> AD – contradictory evidence: no causal links (150, 151)/protective factor (152) - vitamin A/linoleoyl-arachidonoyl-glycerol ratio -> AD (increases risk) (152) - AD -> vitamin A – causal link (vitamin A is decreased in AD – potential biomarker) (152)
Omics biomarkers	Proteomics	- proteins with protective effects against AD: T-cell surface glycoprotein CD5, M-CSF, fractalkine, SLAM, uPA (153), CACYBP, CETN3, MOCS2, TNFAIP8, PVALB (154)
	Metabolomics	- metabolites increasing the risk of AD: phosphate/linoleoyl-arachidonoyl-glycerol ratio, docosatrienoate (155) - metabolites with protective effects against AD: 1-palmitoyl-2-stearoyl-GPC, 1-methylnicotinamide, linoleoyl-arachidonoyl-glycerol, 1-arachidonoyl-GPC (155), histidine (urinary), tyrosine (urinary), alanine (urinary) (156) - metabolites exerting effects on immune cells involved in AD pathogenesis: alpha-ketobutyrate/4-methyl-2-oxopentanoate ratio, CCR2 on monocytes, glycerol/carnitine ratio, HLA DR on CD14-CD16-cells (157)
	Transcriptomics	- a TWAS identified AQP3, PDCD1, ADCY3, DOLPP1 as novel genes involved in AD, encoding measurable serum or tissue proteins (158)
	Lipidomics	- lipid metabolites increasing the risk of AD: TC and CE in very small VLDL; FC in IDL; conc. And PL in medium and large LDL; PL in small LDL; conc., TG, TL, and PL in chylomicrons and extremely large VLDL; linoleic acid/total FA ratio of 18:2 (159); PC (18:1_20:2), PC (O-18:1_20:3) (160); PC (18:0_20:4); PC (18:1_20:4); PC (18:0_20:5); PC (17:0_20:4); SE (27:1/20:4) ratio; SE (27:1/20:5) ratio (161) - lipid metabolites with protective effects against AD: docosahexaenoic acid; arachidonic acid; 1-arachidonoyl-GPE (162); omega-3; omega-6 (163, 164); PC (18:1_20:4); PE (O-18:1_20:4); TAG (56:6); TAG (56:8) (160); PC (16:1_18:1) (161) - AD -> linoleic acid/total FA ratio of 18:2 – causal link (in AD, the ratio is decreased) (159) - AD -> TC, TG, LDL – causal link (decreased levels in AD) (165)
	Immunomics	- cytokines and receptors associated with risk of AD: IL-4, IL-1RA (166), IL-18R1, IL-8, TNFSF14 (153) - cytokines with protective effects against AD: IL-24 (154) - AD -> CXCL5, CXCL10, Axin-1, OSM, SULT1A1, TNFSF14 – causal link (their level is decreased in AD – potential biomarkers) (167)

AD, atopic dermatitis; CE, cholesterol esters; conc., particle concentration; FA, fatty acid; FC, free cholesterol; IDL, Intermediate-Density Lipoprotein; LDL, Low-Density Lipoprotein; PC, Phosphatidylcholine; PE, Phosphatidylethanolamine; PL, phospholipids; Se, selenium; TAG, Triacylglycerol; TC, total cholesterol; TG, triglycerides; TL, total lipids; TWAS, transcriptome-wide association study; VLDL, Very-Low-Density Lipoprotein; Zn, zinc.

Among conventional markers, increased eosinophil and basophil counts, alongside reduced lymphocyte counts, have emerged as risk factors for AD (145), while zinc, selenium, and vitamin E showed consistent protective effects (146, 150, 151). Vitamin D and A yielded contradictory results, with some studies suggesting no causal effect, while others reporting bidirectional associations with AD (147–152). Omics-level studies have managed to considerably expand biomarker discovery, through multi-layered approaches. Proteomic analyses highlighted many protective proteins, such as CD5, M-CSF and fractalkine (153, 154), while metabolomics revealed protective (e.g., phosphate/linoleoyl-arachidonoyl-glycerol ratio, docosatrienoate) or detrimental molecules (e.g., histidine, tyrosine, alanine, 1-methylnicotinamide, glycerophosphocholines), pertaining to their effects on AD (155, 156). Transcriptome-wide association studies identified both established (e.g. FLG) and novel susceptibility genes, such as AQP3 and PDCD1, capable of encoding proteins that could potentially be measured in corporal fluids (158). Lipidomic studies showed that certain lipids, such as specific phosphatidylcholines, conferred risk, whereas omega-3 fatty acids, docosahexaenoic acid (DHA), and several phospholipid or triacylglycerol species were protective against AD (159–164). Immunomic analyses further explored cytokines, associating IL-4, IL-1RA, IL-18R1, and TNFSF14 with an increased AD risk, IL-24 with protective effects, and AD itself with influencing the levels of several cytokines, pointing to downstream biomarkers (153, 166, 167).

Overall, MR-based biomarker discovery in AD has highlighted both classical and omics-derived markers with causal relevance. While conventional measures retain value, multi-omic approaches might offer deeper insights into proteins, metabolites, genes, lipids, and cytokines that might shape the disease risk, or that might significantly be influenced by AD. By integrating diverse omics layers, MR enables the identification of truly causal biomarkers, paving the way for precision medicine and advancing our understanding of immune dysregulation in AD and related complex diseases.

### 3.9 Mendelian randomization in AD therapeutic target identification and drug repurposing

MR has emerged as a powerful tool for identifying and validating therapeutic targets, offering opportunities not only for developing novel treatments, through prioritizing targets with higher likelihood of clinical success, but also for repurposing existing drugs for AD. Moreover, through leveraging information from multi-omics layers, drug targets can be identified more efficiently, offering the potential to transform treatment paradigms in AD. Information from 18 articles using MR methods in order to establish causal inferences with different molecules, in order to uncover potential drug development and repurposing targets, have been summarized in **Table 10** (168–179).

**Table 10:** Summary of the most important findings regarding potential drug targets for treating and preventing AD, and drug repurposing studies, all based on MR.

Drug repurposing/translational targets	Study findings regarding potential drug targets for treating AD
Hypolipemiants	- PCSK9 inhibitors - promising therapeutic agents for AD, not through their hypolipemiant properties, but through their influence on b-NGF (168).
	- another study revealed no causal effects between PCSK9 inhibitors and AD (169).
Angiostatin	- angiostatin - promising therapeutic agent for AD, modulating downstream pathways linked to angiogenesis and inflammation (170).
Proteomics studies for identifying drug targets	- integrating information from a PheWAS with MR methods, 8 proteins were found as drug targets for AD: PVALB, TST (reduced risk of AD), CA14, ECM1, IL22, IL6R, IL18R1, MMP12 (increased risk of AD) (171).
	- a PWAS study has identified 5 drug targets: IL18R1, MMP12, TAPBPL, TLR1, MFNG; IL18R1 and MMP12 are already targeted by drugs which are in clinical trials - potential drug repurposing for AD (172).
	- a multi-omic study, comprising proteomics, metabolomics and immunomics data, identified Il-18R1, CR2, MANSC1, HNRNPAB as potential drug targets for AD (152).
	- UBE2L3 could serve as a translational target for AD, since it exerts an inhibitory role on AD (173).
Transcriptomics studies for drug repurposing and identifying drug targets	- information from a TWAS were integrated with a transcriptome meta-analysis, identifying five potential drug candidates for AD, through leveraging novel genes involved as etiopathogenic factors: pararosaniline (gentian violet derivative), 2-deoxy-D-glucose (2-DG), cantharidin, MG-132, and 1,4-chrysenequinone (174).
	- PCLAF was identified as a potential drug target for AD, through single-cell transcriptome analysis (175).
	- CRAT, TNFRSF6B, ERBB3, IL6R, MMP12, ICAM1, IL1RL2: potential drug targets linked to AD, identified through PWAS and TWAS (176).
	- RARRES2, SERPINC1, GALK1, ECM1: identified as promising novel drug targets for AD (177).
	- IL-13, IL-18R1, TNFSF14, TRANCE (increasing the risk of AD), TNF-β, CD5, CXCL11, IL-33 (decreasing the risk of AD): potential drug targets for treating AD (167).
Metabolomics studies for drug repurposing and identifying drug targets	- S100A12: identified as drug target for AD; its expression is mediated by amino acid metabolites (N6-methyllysine, N2-acetyl,N6,N6-dimethyllysine and N6,N6-dimethyllysine), all linked to risk of AD. Some drugs already target S100A12 and might be of use in AD (e.g. Methotrexate, Rimegepant, Ubrogepant) (178).
	- DHA reduces the risk of AD, potentially through pathways involving tumor necrosis factor ligand superfamily member 14 (TNFSF14), which is negatively correlated with DHA levels, and positively correlated with AD (162–164, 179).
	- 1-arachidonoyl-GPE reduces the risk of AD - promising drug target for preventing and treating AD (162).
	- FADS1, FADS2 genes are involved in lipid metabolism and their expression was associated with decreased risks of AD - therefore, they have been proposed as novel drug targets (161)

AD, atopic dermatitis; MR, Mendelian randomization; PheWAS, phenome-wide association study; PWAS, proteome-wide association; TWAS, transcriptome-wide association study.

Among drug classes, hypolipemiants have drawn attention in regards to AD, since lipids play an important physiopathological role in this inflammatory disease, as they are essential for skin barrier integrity, immune regulation, and inflammation. PCSK9 inhibition was reported as protective against AD, with effects likely mediated through the modulation of β-NGF, rather than the hypolipemiant effects (168), although other studies did not replicate this effect (169). Angiostatin has also emerged as a promising candidate, exerting protective effects through modulating angiogenesis and inflammation (170).

Omics-driven MR approaches, which include proteomics, transcriptomics and metabolomics-derived data, have uncovered a broad range of translational targets. Proteomic MR studies have identified several proteins with therapeutic relevance, such as AD-risk-associated proteins (e.g. IL18R1, MMP12, IL6R, ECM1), protective proteins (e.g. PVALB and TST) (171). Another proteome-wide association study underscored IL18R1 and MMP12 as particularly promising, given that both are already targeted by drugs in clinical development, supporting repurposing potential (172). Multi-omic integration studies further highlighted IL18R1, CR2, MANSC1, HNRNPAB and UBE2L3 as additional candidates (152, 173) also emerged as a protective factor against AD, suggesting translational potential. Transcriptomic approaches have managed to add new layers of evidence regarding more molecules which could potentially aid in treating AD [e.g. pararosaniline, 2-deoxy-D-glucose, cantharidin, MG-132, chrysenequinone (174)]. Many other immune-related proteic targets have been uncovered as potentially useful for AD, such as PCLAF (175), CRAT, TNFRSF6B, ERBB3, ICAM1, IL1RL2 RARRES2, SERPINC1, GALK1, ECM1 (176, 177), reinforcing the transcriptome-proteome overlap in novel drug target discovery. Metabolomic MR studies have also suggested novel translational opportunities. For example, S100A12, a molecule that is regulated by amino acid metabolites, was identified as a druggable protein with existing drugs, such as Methotrexate and Rimegepant, which could be an important drug repurposing trajectory in AD (178). Docosahexaenoic acid (DHA) has also been proven to reduce AD risk through modulation of the TNFSF14 pathways (162–164, 179).

MR studies have revealed multiple druggable pathways in AD in recent years, ranging from cytokine signaling to lipid metabolism. Several proteins and metabolites already overlap with existing drug pipelines, offering immediate repurposing potential, while others represent novel opportunities for translational research. Most importantly, multi-omic MR approaches are able to ensure a more comprehensive map of causal mechanisms, thereby strengthening therapeutic target validation. This integrative strategy enhances the likelihood of clinical success and paves the way for precision medicine in AD and other immune-mediated diseases.

## 4 Benefits and Limitations

There are certain advantages and disadvantages regarding causal inference studies which should be addressed. MR studies have emerged in order to meet the need of clarifying causality, in the context of large amounts of data and correlations produced by GWAS. Even though the latter have provided valuable insights regarding the genetic background of different diseases in the last decades, they are limited by the fact that they focus on the direct analysis of genetic variants, highlighting the most common ones, without providing an in-depth whole-genome analysis, while also relying on imputation techniques for generating results. Recent advances have marked a transition between classic genetics and the modern approach of genomics, which implies a thorough analysis of the whole genome through modern sequencing techniques, providing enormous sets of data, which might pose different challenges for interpretation in the clinical context. However, the existing results of GWAS are not to be ignored, since causal relations between different exposures and outcomes, including AD, are essential for raising hypotheses for conducting complex genomic studies, or even for integrating multi-omic approaches in regards to different diseases.

Therefore, while mentioning the benefits of MR studies, the key strength is the ability to integrate the genetic variants and associations discovered through GWAS in clinical context, strengthening causal inferences, in order to raise pertinent premises for large-scale studies. MR is also able to overcome the challenges posed by conventional epidemiology, such as confounding factors or reverse causation, while also sometimes taking the place of randomized control studies, when these are not feasible (13, 15). Regarding AD, MR serves as a powerful tool to clarify the molecular basis of this disease, and to discover potential biomarkers or novel drug targets, in order to enable more personalized and effective treatment approaches.

Even though the benefits of MR remain substantial, certain challenges are posed along the way. Some of them arise from horizontal pleiotropy, where a genetic variant affects the outcome through biological pathways which are unrelated to the exposure. In order to overcome this challenge, specialized sensitivity analyses have been developed for making necessary corrections. Many MR studies are also subjected to variability due to different statistical methods and assumptions that are used (e.g. inverse-variance weighted, MR-Egger regression, weighted median and mode-based estimators, or multivariable MR). There is also a considerable influence exerted by the GWAS results that are used for MR analyses, both in a quantitative and a qualitative manner. Quantitatively, the size of the GWAS datasets can influence the overall analysis, since small amounts of data might lead to unreliable results. Qualitatively, the diversity of the chosen GWAS cohorts holds an important role for generalizing the results, since cohorts consisting of various ancestries can increase external validity, minimizing health inequities across populations (15). Unfortunately, most GWAS have been primarily conducted on populations of European descent, which could subject MR studies to bias. However, GWAS regarding AD have favorably targeted diverse populations and ancestries (European, North American, Australian, Chinese Han, Japanese, Korean, African), uncovering general and population-specific genetic variants analyzed through MR.

## 5 Discussion and conclusion

Mendelian randomization is a powerful tool for disentangling the causal relationships between atopic dermatitis and many different exposures and outcomes, extending knowledge beyond traditional epidemiological and observational associations. MR studies have helped clarify etiopathogenic pathways and comorbidities, uncovering risk factors and protective exposures, while also identifying conventional and novel, omics-derived biomarkers. Crucially for the clinical setting, they have highlighted novel therapeutic targets and drug repurposing opportunities. Different omics layers, such as genomics, epigenomics, transcriptomics, proteomics, metabolomics, lipidomics, and microbiomics, are able to generate valuable candidate translational targets. Unfortunately, limited efforts have been made in order to integrate these datasets, or to explore their overlap, contributing to variability and fragmentation of findings. Multi-omic approaches, including platforms specifically designed to integrate diverse omics data [e.g., PlatOMICs (180)], are therefore crucial for mapping the complex molecular interactions underlying AD. Looking forward, coupling multi-omics MR with advanced computational methods, such as AI-driven image analysis, could better connect molecular signatures in the clinical context, accelerating the discovery of novel diagnostic and prognostic biomarkers and drug targets for AD.

There are still a few caveats regarding MR studies that should be overcome in the future, in order to maximize the benefits of this statistical method. For example, trans-ethnic MR validation is a pressing matter that will be facilitated through the growing availability of GWAS data across diverse populations, improving population representativeness. Multivariable Mendelian Randomization (MVMR) addresses the need for multivariable MR combining multiple exposures, overcoming the limitation of traditional MR, which estimates causal effects in regards to a single exposure. To conclude, MR studies have already provided causal insights across multiple biological layers in AD, but their true potential lies in multi-omic integration. By combining MR with machine-learning-driven omics integration, future studies may enable causal networks for precision dermatology. Such strategies are poised to transform patient care, paving the way for reliable biomarkers, personalized prevention, and more effective, targeted therapies.
